# Optimizing Images for an E-Cigarette Messaging Campaign: Liking and Perceived Effectiveness

**DOI:** 10.3390/ijerph182412989

**Published:** 2021-12-09

**Authors:** Elise M. Stevens, Brittney Keller-Hamilton, Darren Mays, Jennifer B. Unger, Olivia A. Wackowski, Julia C. West, Andrea C. Villanti

**Affiliations:** 1 Department of Population and Quantitative Health Sciences, Division of Preventative and Behavioral Medicine, University of Massachusetts Medical School, Worcester, MA 01605, USA; 2 Center for Tobacco Research, The Ohio State University Comprehensive Cancer Center, Department of Internal Medicine, College of Medicine, The Ohio State University, Columbus, OH 43214, USA; brittney.keller-hamilton@osumc.edu (B.K.-H.); darren.mays@osumc.edu (D.M.); 3 Department of Population and Public Health Sciences, University of Southern California, Los Angeles, CA 90032, USA; unger@usc.edu; 4 Center for Tobacco Studies, Department of Health Behavior, Society and Policy, Rutgers School of Public Health, Rutgers University, New Brunswick, NJ 08854, USA; wackowol@cts.rutgers.edu; 5 Department of Psychological Science, University of Vermont, Burlington, VT 05405, USA; julia.west@uvm.edu; 6 Vermont Center on Behavior and Health, Department of Psychiatry, University of Vermont, Burlington, VT 05405, USA; andrea.villanti@uvm.edu

**Keywords:** health communication, message testing, electronic vapor products

## Abstract

Introduction: Given the prevalence of electronic vapor product (EVP) use among young people in the US, there is a need for effective vaping education campaigns. This study tested 32 images for liking and perceived effectiveness (PE) to identify optimal images for a messaging campaign. Method: Images were selected from current campaigns, warning labels, and other images based on young adult reasons for use. Images were coded for the presence of (1) people, (2) vapor, (3) device, (4) color, and (5) similarity to warning label image. Young adults (*n* = 200) were recruited from the Amazon Mechanical Turk platform. Participants were randomly assigned to view and rate six of the 32 images on liking as well as PE, which measured the potential impact of the image to discourage vaping appeal and use. Results: Images containing vapor and/or a device or e-liquid were not well-liked but were perceived as effective in discouraging vaping (ps < 0.05). Images from warning labels were also not well-liked but were perceived as significantly more effective than those not from a warning (*p* < 0.01). Liking and effectiveness of features was similar for both EVP users and non-users. Discussion: Images with specific features were rated as less likable but rated as higher on PE. However, the consistency of image features rated as effective by EVP users and non-users supports the utility of similar imagery for vaping prevention and reduction efforts.

## 1. Introduction

Targeted digital media campaigns have emerged as a common strategy for tobacco prevention [1,2,3,4]. Given ongoing health concerns and the rise in vaping among young adults (YAs) [5,6], we developed vaping prevention social media messages [7] due to the US Food and Drug Administration’s initiative to reduce young adult vaping [8]. Formative message testing is an essential part of the message development process. For instance, in a previous study, we found that few vaping prevention message features were rated as likable and effective by YAs. In advertising for consumer products, message likeability predicts advertising success [9,10], and perceived effectiveness (PE) indicates how well the message targets campaign-related beliefs and attitudes [11,12,13]. This study builds on our previous formative work by evaluating the role of the images themselves in liking and PE [7]. Developing and selecting images for vaping prevention campaigns can be challenging because program planners may need to balance selecting features that may attract attention (which may include features that are likable) and show product imagery, with the potential that such features could unintentionally reinforce the appeal of vaping. Thus, consideration of the balance and tradeoffs of participant ratings such as likability and PE may be informative. Image testing alone without text was chosen due to the increased use of picture-based social media (e.g., Instagram) among young adults [14] and to contribute to the limited literature based on potentially effective imagery for vaping prevention communication interventions. Based on findings from previous studies, we expected that images similar to those used in a warning label study [15] and those with people would have higher PE for prevention [16]. We also expected that images with people, color, devices or e-liquid, and vapor would be more likable due to their reported high level of appeal in other studies [16,17]. For instance, prior research has shown that more attention is paid by young adults to the models in EVP advertisements than other features [16]. In addition, another study found that vapor was also an alluring aspect of advertisements for EVPs [17].

## 2. Materials and Methods

### 2.1. Sample, Procedures, and Measures

In March 2020, we conducted an online survey of YAs ages 18 to 24 (*n* = 200) on Amazon’s Mechanical Turk (MTurk). Participants were paid $1.00. After consenting, participants completed sociodemographic and tobacco use questions (including ever and past 30-day electronic vapor product (EVP) use), then were randomized to see six of 33 text-based messages and six of 32 images. Results of the text messages have been described elsewhere [7]; the results of this study focus on the image ratings. Participants saw six images which allowed for adequate viewing numbers for each image while limiting the participant burden. After each image, participants rated the image for likeability (on a five-point scale from “I disliked it very much” to “I liked it very much.” [9,10]) and prevention-related perceived effectiveness (PE). PE was assessed with three questions adapted from a validated scale [18]: This image (1) “discourages me from wanting to vape”, (2) “makes me concerned about the health effects of vaping,” and (3) “makes vaping seem unpleasant to me” on a five-point scale ranging from “strongly disagree” to “strongly agree” [18]. Responses to the three items for each participant were averaged for each image they viewed. These data are from a larger study examining content to reduce vaping [7]. Procedures were approved by the University of Vermont IRB.

### 2.2. Images and Coding

Images (*n* = 32) were chosen from Unsplash, a website that houses free stock photography, based on young adult reasons for use (e.g., socializing, flavors) [19,20,21,22] and to be similar to those appearing in prevention campaigns, including those in the United States and United Kingdom, and in a warning label study [23]. Two coders coded all images independently using a codebook developed a priori based on prior research. The coders reached 100% reliability in coding images for: (1) multiple and bright colors present [24], (2) a device or e-liquid container, (3) people—including body parts, (4) vapor, and were (5) similar to those used in an EVP warning label study [23]. No images contained text.

### 2.3. Data Analysis

We calculated mean likeability and PE for each image (Table 1) and assessed the association between the two using a linear mixed-effects model with restricted maximum likelihood (REML). Univariable (one feature) and multivariable (all features) using a mixed-effects model with REML examined the effect of features on likeability and PE, accounting for multiple observations within subjects. Additional models assessed interactions between EVP use and image features.

## 3. Results

### 3.1. Participants

Participants were largely male (70%), white (60%) and past 30-day EVP users (64%; *n* = 128). Full sample characteristics are described elsewhere [7]. Overall, 19 (59%) of the images were coded as colorful, 7 (22%) featured a device or e-liquid, 20 (63%) included people, 12 (38%) featured vapor, and 4 (13%) were similar to images in a warning label study. Generally, image likeability and PE were inversely correlated, such that a 1-point increase in mean likeability was associated with a 0.22 decrease in mean PE (95% CI: −0.27, −0.18).

### 3.2. Image Features: Liking

Mean likeability ranged from 2.39 (Image 18: people, vapor) to 3.97 (Image 2; color; Table 1). Univariable results identified color as the only image feature that was positively associated with likeability, but that was attenuated in the multivariable model (Table 2). Univariable and adjusted results showed that images with the presence of people, vapor, devices or e-liquids, and images from warning labels had lower mean likeability than images without those features. The models testing all image features and including interactions with EVP use status was only significant for not past 30-day users, finding that the presence of a device or e-liquid was less likable in the images than those without it.

### 3.3. Image Features: Perceived Effectiveness

Mean PE ranged from 1.94 (Image 2; color) to 3.40 (Image 32; Table 1; warning). In the univariable and adjusted analysis, images with the presence of vapor and those from a warning label study were rated as more effective than images without those features. The presence of a device/e-liquid was also positively associated with PE in the adjusted model (Table 2).

The models testing interactions between image features by EVP use status showed that presence of vapor was seen as more effective for non-users (Coeff. = 0.60, *p* < 0.001) and users (Coeff. = 0.27, *p* < 0.001). The presence of color was seen as less effective for non-users only (Coeff. = −0.23, *p* < 0.01).

## 4. Discussion

Image features rated as effective in discouraging vaping in YAs (i.e., presence of vapor, device/e-liquid, warning images) were rated as less likable than images without those features. In models adjusting for all image features, color was not associated with likeability or PE and the presence of people reduced the likeability of images with no effect on PE, contrary to our expectations which were supported by prior research on EVP messaging appeal [16,17,25]. Results were comparable in past 30-day EVP users and non-users, supporting the utility of similar imagery in vaping prevention and reduction efforts. These results differed from our message-testing in the same sample, in which the likeability and perceived effectiveness of text-only messages were positively correlated [7]. Our findings suggest that the inclusion of some features like color or vapor may only signal one of the two motivational systems (i.e., appetitive and aversive) when a balance of the two might be best for health communication [25]. For instance, it could be that features that are well-liked by young adults may not actually be the images that are effective in preventing vaping. In a past study, young adults found models in EVP advertisements more attention-grabbing than other features [16]. This may not translate to messages aimed at preventing vaping. Health communicators should consider the differences between liking and PE when pre-testing messages for campaigns. In addition, health communicators also might consider that effective imagery may be the same for EVP users and non-users.

This study has several limitations. First, the sample was drawn from MTurk and included a high proportion of EVP users. MTurk has been used in other tobacco surveys [26] with survey samples that are comparable to experimental and population samples, [27], but our findings may not be generalizable. Second, the study only tested 32 images and five features in a relatively small sample and there could be many more features that impact liking and PE. In addition, there was an unequal distribution of features within the messages and many of the images were stock photos not specifically designed for EVP messaging. Third, since measures of liking are often borrowed from the advertising literature [28], it may be that this measure does not always translate to health education campaigns. Finally, the application of a PE scale to assess image effectiveness was novel and not well-established in the literature. Future studies should explore the usefulness of these images using other established measures as well, including attention.

## 5. Conclusions

Our prior study used the overall likeability and PE ratings to determine the images to be paired with vaping-related messages in a subsequent trial; results from that study suggested that the images had no effect on the overall likeability or effectiveness of the message/image pairs in a separate sample of YAs [7]. The current analyses, however, highlight that different images may have been selected for inclusion in our message optimization trial, based on the specific features of the images deemed to be effective in discouraging vaping, rather than the overall ratings. The findings highlight common elements of imagery likely to be effective in reducing vaping in YAs, specifically warning images. Future studies addressing a greater range of image features are needed to optimize the images used in vaping prevention campaigns and determine whether and how these features enhance their effectiveness.

## Figures and Tables

**Table 1:** Images, coding, likeability, and perceived message effectiveness.

Image	Image Assigned#	Coding	*n*	Likeability M(SD)	PME M(SD)
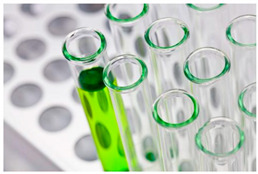	1	C, W	39	3.03 (1.16)	2.41 (1.35)
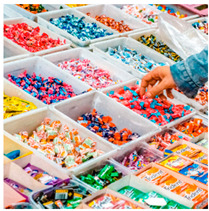	2	C	39	3.97 (0.81)	1.94 (1.17)
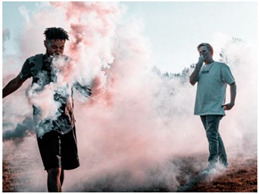	3	C, P, V	38	2.74 (1.27)	3.32 (1.30)
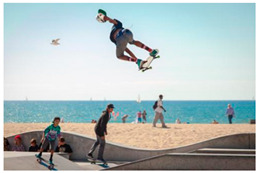	4	C, P	38	3.55 (0.95)	2.27 (1.24)
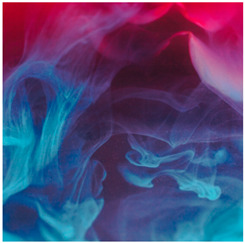	5	C, V	37	3.27 (1.22)	2.77 (1.32)
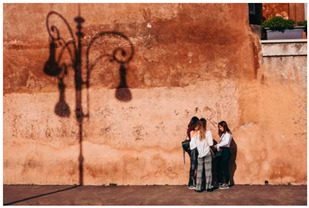	6	C, P	36	3.06 (0.83)	2.59 (1.17)
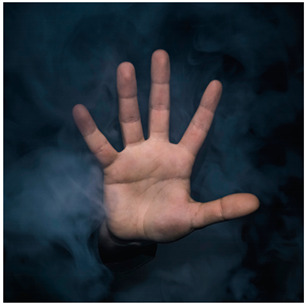	7	P, V, W	38	3.11 (1.06)	3.16 (1.20)
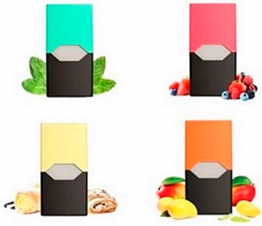	8	C, D	36	3.06 (1.07)	2.17 (1.19)
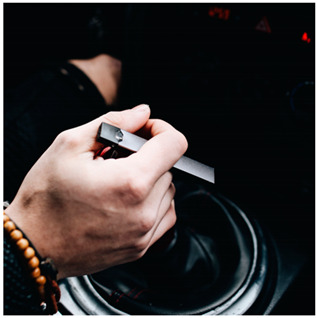	9	D, P	38	2.74 (1.08)	2.36 (1.27)
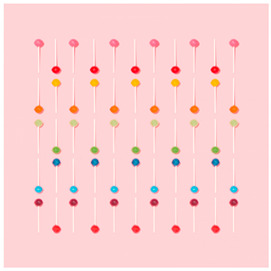	10	C	38	3.61 (1.10)	2.54 (1.36)
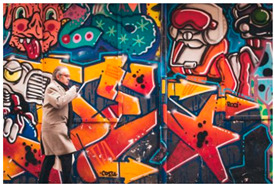	11	C, P	38	3.08 (0.88)	2.44 (1.28)
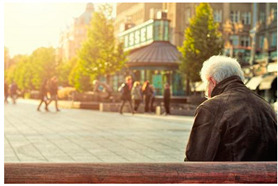	12	C, P	38	3.11 (1.11)	2.29 (1.35)
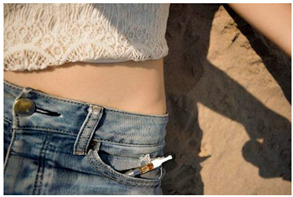	13	C, D, P	37	2.62 (1.09)	2.38 (1.22)
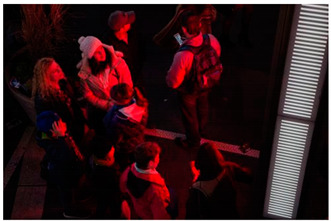	14	P	36	3.19 (0.82)	2.83 (1.18)
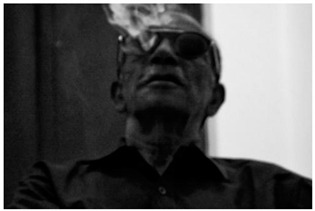	15	P, V	36 ^a^	2.43 (1.07)	2.92 (1.21)
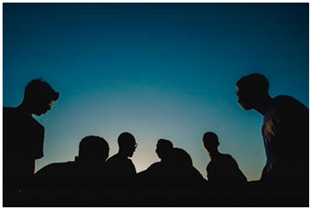	16	P	38	3.39 (1.08)	2.77 (1.44)
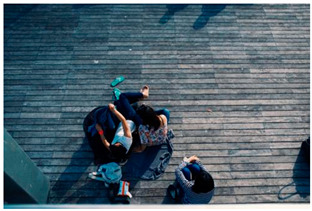	17	C, P	38	3.16 (1.17)	2.49 (1.37)
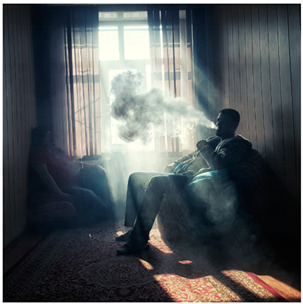	18	P, V	36	2.39 (1.10)	3.19 (1.08)
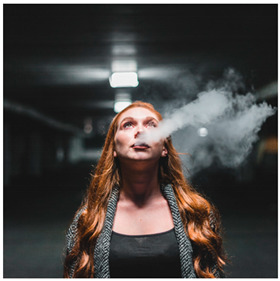	19	P, V	39 ^a^	3.05 (1.11)	2.84 (1.41)
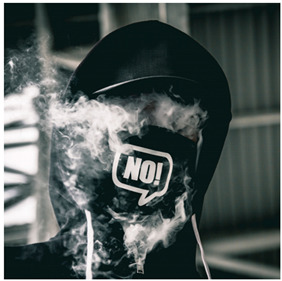	20	P, V	37	2.86 (1.23)	3.07 (1.24)
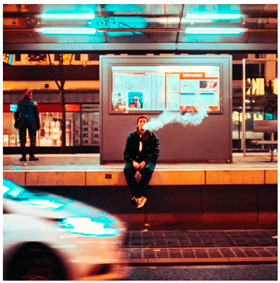	21	C, P, V	37	3.27 (1.15)	2.51 (1.31)
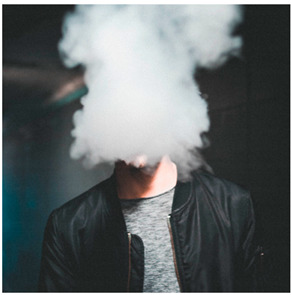	22	P, V	35	2.66 (1.28)	2.78 (1.25)
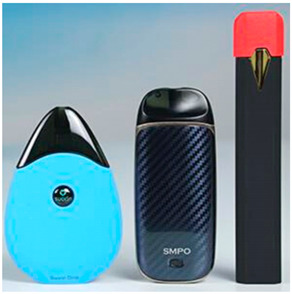	23	C, D	36	3.28 (1.06)	2.92 (1.22)
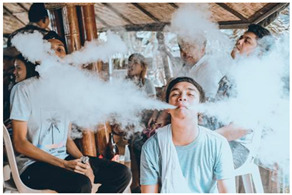	24	C, P, V	38	2.63 (1.32)	2.97 (1.32)
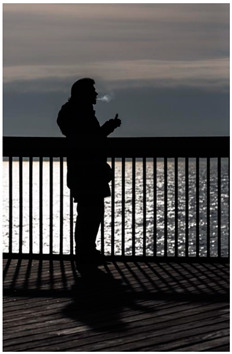	25	D, P	37	3.14 (1.25)	2.44 (1.31)
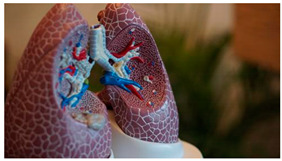	26	C, W	38	2.63 (1.10)	2.75 (1.30)
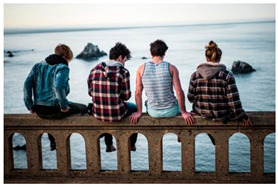	27	C, P	38	3.58 (1.00)	2.10 (1.32)
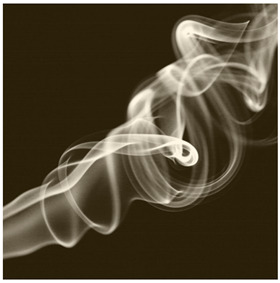	28	V	38	3.08 (1.15)	2.76 (1.32)
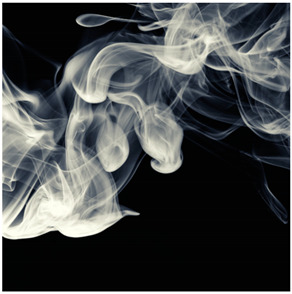	29	V	37 ^a^	3.17 (1.23)	2.53 (1.18)
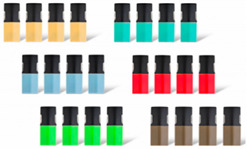	30	C, D	36	2.97 (1.21)	2.76 (1.34)
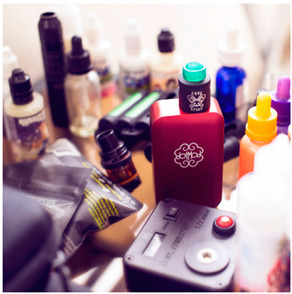	31	C, D	38	2.63 (1.08)	2.91 (1.29)
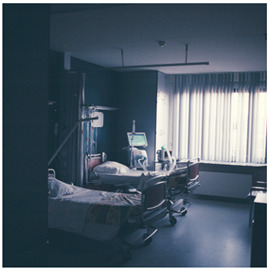	32	W	38	2.47 (1.29)	3.40 (1.21)

Note: Images (*n* = 32) were chosen from Unsplash, a website that houses free stock photography, based on young adult reasons for use (e.g., socializing, flavors) [19,20,21,22] and to be similar to those appearing in prevention campaigns including those in the United States and United Kingdom, and in a warning label study [23]. C, use of multiple and bright colors; D, actual device or e-liquid container present; P, presence of people—including body parts; V, presence of vapor or vapor clouds; W, similar to images used in a warning label study. ^a^ Missing *n* = 1 for likeability.

**Table 2:** Univariable and Multivariable Models Estimating Image Features’ Associations with Likeability and Perceived Effectiveness ^a^.

	Likeability		PE	
	M (SE)	*p*-Value	M (SE)	*p*-Value
**Univariable models**				
People (vs. no people)	−0.13 (0.06)	0.03	0.04 (0.05)	0.36
Vapor (vs. no vapor)	−0.19 (0.06)	0.001	-	-
Past 30-day EVP users	-	-	0.21 (0.06)	<0.001
Not past 30-day EVP users	-	-	0.52 (0.08)	<0.001
Device/e-liquid (vs. no device/e-liquid)	-	-	−0.06 (0.06)	0.29
Past 30-day EVP users	0.05 (0.08)	0.56	-	-
Not past 30-day EVP users	−0.48 (0.11)	<0.001	-	-
Color (vs. predominantly black/dark)	0.23 (0.06)	<0.001	-	-
Past 30-day EVP users	-	-	−0.15 (0.06)	0.01
Not past 30-day EVP users	-	-	−0.40 (0.08)	<0.001
Warning (vs. not similar to image from a warning)	−0.37 (0.08)	<0.001	0.35 (0.07)	<0.001
**Multivariable models**				
People (vs. no people)	−0.26 (0.06)	<0.001	0.08 (0.05)	0.12
Vapor (vs. no vapor)	−0.32 (0.07)	<0.001	-	-
Past 30-day EVP users	-	-	0.27 (0.07)	<0.001
Not past 30-day EVP users	-	-	0.60 (0.08)	<0.001
Device/e-liquid (vs. no device/e-liquid)	-	-	0.23 (0.06)	<0.001
Past 30-day EVP users	−0.28 (0.09)	0.003	-	-
Not past 30-day EVP users	0.80 (0.12)	<0.001	-	-
Color (vs. predominantly black/dark)	0.05 (0.06)	0.473	-	-
Past 30-day EVP users	-	-	0.04 (0.06)	0.56
Not past 30-day EVP users	-	-	−0.23 (0.08)	0.004
Warning (vs. not similar to image from a warning)	−0.64 (0.09)	<0.001	0.49 (0.07)	<0.001

Abbreviations: M = mean; SE = standard error; PE = perceived effectiveness; EVP = electronic vapor product. Electronic vapor product (EVP) use was categorized as the following: participants who reported that they had used EVP in the past 30 days were categorized as current users, and participants who reported never using e-cigarettes, or formerly using e-cigarettes, were categorized as non-users. ^a^ Mixed-effects linear regression models with random intercepts were used to estimate the effect of feature inclusion on image likeability and PE. Models analyzed the interaction between EVP use status, but results are only stratified by EVP use status when the interaction was statistically significant. Stratified results are presented from models with statistically significant interactions between the image component and EVP user status.

## Data Availability

Data available upon request.
